# The Development of Digital Image Colorimetric Quantitative Analysis of Multi-Explosives Using Polymer Gel Sensors

**DOI:** 10.3390/s21238041

**Published:** 2021-12-01

**Authors:** Yudtapum Thipwimonmas, Adul Thiangchanya, Apichai Phonchai, Sittipoom Thainchaiwattana, Wachirawit Jomsati, Sunisa Jomsati, Kunanunt Tayayuth, Warakorn Limbut

**Affiliations:** 1 Division of Health and Applied Sciences, Faculty of Science, Prince of Songkla University, Hat Yai, Songkhla 90110, Thailand; t.yudtapum@hotmail.com (Y.T.); adul.t@psu.ac.th (A.T.); papichai_13@hotmail.com (A.P.); 2 Forensic Science Innovation and Service Center, Prince of Songkla University, Hat Yai, Songkhla 90110, Thailand; 3 Police Forensic Science Center 9, M.1, Chalung, Hat Yai, Songkhla 90110, Thailand; 091sittipoom@gmail.com (S.T.); jomsati@gmail.com (W.J.); tukly.1986@gmail.com (S.J.); 4 Science Park, Hat Yai Campus of Extension Southern Institute of Science Park, Prince of Songkla University, Moo 6, Hat Yai, Songkhla 90110, Thailand; Kunanunt@gmail.com; 5 Center of Excellence for Trace Analysis and Biosensors (TAB-CoE), Prince of Songkla University, Hat Yai, Songkhla 90110, Thailand; 6 Center of Excellence for Innovation in Chemistry, Faculty of Science, Prince of Songkla University, Hat Yai, Songkhla 90110, Thailand

**Keywords:** multi-explosives, digital images, RGB intensity values, 96-well plates, colorimetric detection, polymer gel sensor

## Abstract

Polymer gel sensors on 96-well plates were successfully used to detect four different multi-explosives, including 2,4,6-trinitrotoluene (TNT), 2,4-dinitrotoluene (DNT), nitrite, and perchlorate. The products of reactions between the explosives and the polymer gel sensors were digitally captured, and the images were analyzed by a developed Red–Green–Blue (RGB) analyzer program on a notebook computer. RGB color analysis provided the basic color data of the reaction products for the quantification of the explosives. The results provided good linear range, sensitivity, limit of detection, limit of quantitation, specificity, interference tolerance, and recovery. The method demonstrated great potential to detect explosives by colorimetric analysis of digital images of samples on 96-well plates. It is possible to apply the proposed method for quantitative on-site field screening of multi-explosives.

## 1. Introduction

Explosions can inflict serious, even life-threatening injuries on many people at the same time [1]. Explosions cause damage in several different ways: by the blast wave, the shock wave, heat, fragmentation, or the blast wind. Explosives are commonly classified as high and low explosives, according to the type and velocity of the reaction involved [2]. Homemade explosive devices or improvised explosive devices (IEDs) are simple to make using widely accessible materials. The increased controls placed on commercial and military explosives has made the use of IEDs more prevalent. IEDs can vary widely in size; most have at least four components: a power source, a main charge, an initiator, and a switch. In addition, a casing or container may be used, as well as a booster and added fragmentation or shrapnel, such as nails or screws [3]. The use of IEDs has increased dramatically in recent years in the southern border region of Thailand. In Yala, Pattani, Narathiwat provinces, and some districts of Songkhla province, violent conflict has frequently involved the use of IEDs.

Terrorist activities have generated an enormous demand for the rapid identification of explosive compounds at the site of the terrorist act. Builders of IEDs will modify a device to efficiently use the available materials, and a mix of high explosives and low explosives may be used. The available data may help to identify a terrorist group or bomber by linking the explosive evidence to known designs or materials used in previous explosions [4]. Field analysis of explosives needs a rapid, portable, real-time, low-cost, and reliable method of identification. The need for quantification of trace explosives has become a matter of urgency due to the increasing threat from terrorist attacks [5].

A variety of detection methods for the quantitative analysis of explosives has been reported. The instrumental techniques used include gas sensors [6,7,8,9,10], high-performance liquid chromatography [11,12], capillary electrophoresis [13,14], ion chromatography [15,16], and electrochemistry [17,18]. However, some of these methods are typically laboratory-based, time-consuming, require sample extraction and preparation, and require expert knowledge for effective results.

In recent years, rapid quantification by colorimetric analysis of digital images has received growing interest. This method has been used for the quantitative analysis of explosives [19,20,21] and other applications [22,23,24]. The method is based on the measurement of the intensity of red, green, and blue (RGB) basic color data in a digital image of a reaction product. The RGB intensity values obtained from a color image can be used to generate calibration equations for quantitative analysis. The colorimetric method is an interesting approach because it is inexpensive and does not require expert analysis. For example, the development of rapid quantitative determination of 2,4,6-trinitrotoluene (TNT) and ammonium nitrate explosives made use of a software application installed on a mobile phone [25,26]. The response was fast, processing was possible in real time, the procedure was simple, and the device was portable. Nevertheless, only one sample could be analyzed at a time. When a large number of samples had to be analyzed, the process took a long time, but the rapid quantitative analysis of multiple samples has recently been reported. A microplate spectrophotometer was used to analyze samples on 96-well microplates, which could simultaneously analyze many samples with high accuracy, but the equipment was both large and laboratory-based.

Polymer gel materials, which are composed of long macromolecular chains with a 3D cross-linked structure, have been increasingly investigated [27,28,29,30]. Sodium carboxymethyl cellulose (CMC) is a kind of high-polymer cellulose ether with properties of stability, water absorption, and thickening, bulking, and gelation abilities [31], whereas polyethylene glycol (PEG) can be used as a plasticizer that is soluble in water and many organic solvents. PEG accelerated the normal gelation process and was approved as a nontoxic product by the U.S. Food and Drug Administration [32,33]. The porous network of a polymer gel could be used to entrap reagents to produce a polymer gel sensor for the detection of explosives. Then, the colorimetric products of the reaction within the polymer gel sensor can be placed on 96-well plates and processed into digital images for analysis. An RGB analyzer program can measure the intensity of red, green, and blue (RGB) color values from the digital images, which can provide a simple quantification.

This work describes the development of a new, simple, and rapid field analysis method using 96-well plates containing sensors based on a binary polymer composite gel entrapping appropriate reagents. The work consisted of two parts. Firstly, we designed and fabricated a photographic box and created a software program for the analysis of RGB color intensity of the digital images and studied the analytical performance of the proposed method. Secondly, we applied the method for the high-throughput determination of multi-explosives. Digital image colorimetric analysis was applied for the determination of 2,4,6-trinitrotoluene (TNT), 2,4-dinitrotoluene (DNT), nitrite, and perchlorate, which have been used in the three southernmost provinces of Thailand [4]. The portable method demonstrated rapid, accurate, and real-time simultaneous quantitative analysis of multi-explosives.

## 2. Materials and Methods

### 2.1. Chemicals and Materials

Sodium carboxymethyl cellulose (CMC, Mw ≈90,000 g mol^−1^), 2,4,6-trinitrotoluene (TNT, 96%), and 2,4-dinitrotoluene (DNT, 97%) were purchased from Sigma-Aldrich (Steinheim, Germany). Sodium nitrite (NaNO_2_, ≥97.0%) was analytical grade from Ajax Finechem (Auckland, New Zealand). Potassium hydroxide (KOH, ≥85.0%), potassium iodide (KI, ≥99.5%), methylene blue (≥82%), sulfuric acid (H_2_SO_4_, 95.0–97.0%), ethanol (≥99.9%), and acetone (≥99.8%) were of analytical grade, and all were purchased from Merck KGaA (Darmstadt, Germany). Potassium perchlorate (KClO_4_, ≥99%) was from Sigma-Aldrich (St. Louis, MO, USA). All chemicals were analytical grade. Deionized water was used for the preparation of all solutions (18.2 MΩ∙cm) (Barnstead^TM^ Easy Pure^TM^ II water purification system, Thermo Fisher Scientific^TM^, Marietta, OH, USA). A UV/Vis microplate spectrophotometer (Thermo Fisher Scientific, Model 51119200, Multiskan^TM^ GO, Vantaa, Uusimaa, Finland) was used for absorbance measurements. Clear polystyrene (PS) 96-well Microwell (Thermo Scientific Nunc^®^, Part No: NAL-167008, Suzhou, Jiangsu, China) was used for the preparation of the polymer gel sensor.

### 2.2. Preparation of Polymer Gel Sensor

Sodium carboxymethyl cellulose (CMC) was dissolved in distilled water (2.5% *w*/*v*), Then, 2 mL of polyethylene glycol (PEG-400) was added, and the mixture solution was magnetically stirred for an hour at room temperature. A transparent viscous gel was obtained. The polymer gel sensors for the determination of each explosive were prepared by mixing polymer gel and the appropriate reagent in a 1:1 ratio under stirring for 30 min. Then, the wells of 96-well plates were filled with 200 µL of the sensor mixture, and the plates were kept at room temperature overnight. During the gelling process, the reagents were entrapped inside the pores of the polymer gel.

### 2.3. Color Test of Explosives

First, 2,4,6-trinitrotoluene (TNT) and 2,4-dinitrotoluene (DNT) were prepared with 1:1 acetone–water [25,34]. TNT concentrations were in the range of 0.250 to 100 mg L^−1^ and DNT concentrations were in the range of 0.025 to 0.90 mg mL^−1^. One hundred µL of TNT and DNT standard solutions of each concentration were tested. The polymer gel sensor was prepared by mixing the polymer gel with 0.25 mol L^−^^1^ of potassium hydroxide in ethanol (1:1 ratio). This reagent changed the colorless sensor to a red-brown color in the presence of TNT and a blue color in the presence of DNT.

To test nitrite, a range of nitrite standard solutions (0.25–80 mg L^−1^) were prepared in deionized water, and 100 µL of each nitrite standard was tested with a sensor of polymer gel mixed with the two reagents (0.07 mol L^−1^ sulfuric acid and 0.07 mol L^−1^ potassium iodide) in a 1:1 ratio. The colorless gel sensor turned yellow in the presence of nitrite. To test perchlorate, standard perchlorate solutions were prepared in deionized water to appropriate concentrations in the range of 0.0050 to 1.0 mg mL^−1^. The polymer gel was mixed with methylene blue (MB) at 0.025% *w*/*v* in a ratio of 1:1, which turned violet in the presence of perchlorate. 

All test samples were left in the 96-well plates for 5 min prior to observation of the resultant color. Each color test was repeated three times. The RGB intensity of the colored products was detected while the samples were on the 96-well plates using the RGB analyzer program (PSU RGB Analyzer Plus 3.0) on a notebook.

### 2.4. Photographic System

**Figure 1:**
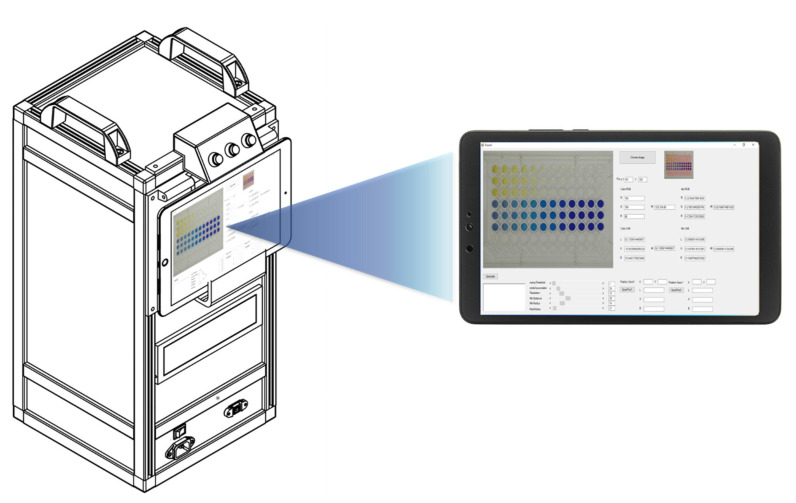
RGB analysis system for the determination of multi-explosives.

A photographic box measuring 24.0 cm × 29.0 cm × 53.0 cm was designed and fabricated from opaque black acrylic sheet with a white internal background (Figure 1). The photographic box was equipped with a camera, power supply, battery, two microcontrollers, and two motors. Twelve white high-intensity light-emitting diodes (LEDs) were used as the light sources inside the box. The images were captured 5 min after the addition of the standard/sample solution. The images were captured with an industrial video camera (ELP USB 8MP high-resolution SONY IMX179) within the photographic box. Each image was 64.0 KB (640 × 480 pixels) in size and was saved as a JPEG (24-bits) on a 2-in-1 notebook in tablet mode (Acer One 10, HD (1280 × 800), IPS).

Once the images were captured, they were automatically transferred to the computer with the program command. The RGB values of the colorimetric product were analyzed with the in-house designed RGB analysis program. Each colorimetric test was repeated three times from three wells of the microplate. The average intensities of the red, green, blue, and total RGB (T; T = R + G + B) from the three replications were used as single data points in graphs.

**Figure 2:**
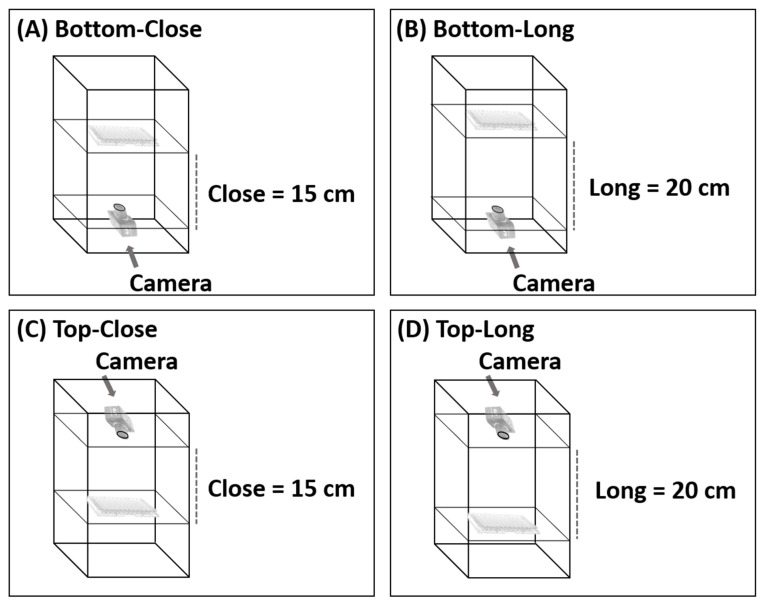
Various configurations of the photographic system; (**A**) Bottom-Close, (**B**) Bottom-Long, (**C**) Top-Close and (**D**) Top-Long.

To find the best camera position in the photographic system, images were captured from above and below the 96-well plates and from a distance of 15 cm and 20 cm (Figure 2). If the distance was less than 15 cm, the camera could not focus. Various conditions were optimized. The condition considered optimal was that which produced the lowest relative standard deviation (%RSD) of different concentrations (20, 40, 60, 80, and 100 mg L^−1^) of four food colorings (red, green, yellow, and black). To study the precision of the proposed method, entire 96-well plates of each food coloring were analyzed at a concentration of 80 mg L^−1^ (n = 96), and the results were compared with the results obtained from analysis with the spectrophotometer.

### 2.5. The RGB Analyzer Program

**Figure 3:**
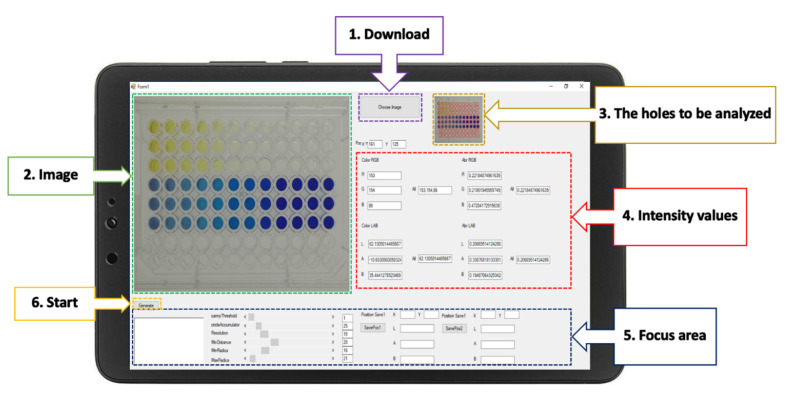
The graphical interface of the RGB analyzer program for the determination of multi-explosives.

The RGB analyzer program was designed for multi-explosives determination. This program consists of six functions, and the interface presents six elements (Figure 3): the download command to input the images to the program; the image presented after download; a screen that shows the wells to be analyzed; the intensity values displayed in real time; a focus adjustment for better analysis of a specific area; and the start command of the RGB analyzer program. After the start command has been initiated, the analytical data will automatically appear in the Excel program. Data can be transferred to connected colleagues on a network system or by Bluetooth.

### 2.6. Analytical Performance and Method Validation

The linear range, limit of detection (LOD), precision, accuracy, interference effect, and specificity of the method were investigated. The LOD was calculated by using LOD = 3(S_a_/b), where S_a_ is the standard deviation of the intercept and b is the slope of the calibration curve. Precision was indicated by the relative standard deviation percentage (%RSD) for each RGB value. The accuracy of the method in terms of percent recovery was evaluated by analyzing the known concentration of each explosive standard solution against the established calibration curve; specificity was tested toward each explosive singly and toward mixtures of two, three, and four explosives.

### 2.7. Interference Effects

Next, we assessed the specificity of the proposed method for the measurement of the studied explosives. The interference effects of the developed method for multi-explosives determination were investigated by testing some species (urea, Zn^2+^, Ba^2+^, Cu^2+^, Pb^2+^, Ni^2+^, Mg^2+^, Fe^2+^, Sb^3+^, SO_4_^2−^, Cl^−^, and NO_3_^−^) that were previously reported to be present in real samples [18,21,35]. The tolerance limits for the potential interfering substances were defined as the maximum concentration that produced a signal error less than ±5% compared with the signal from standard explosives determined by mixing different concentrations of these possible interference species with 10 mg L^−1^ TNT, 0.5 mg mL^−1^ DNT, 2 mg L^−1^ nitrite, and 0.7 mg mL^−1^ perchlorate under the optimal conditions.

### 2.8. Application for Multi-Explosives Sensing

To simulate the forensic conditions encountered in real-case scenarios, six different material samples were used as substrates during the collection of explosive residues. These materials were a table, a tile floor, a mobile phone, a windowsill, a PVC pipe, and a wall clock. The samples were collected from substrate surfaces by swabbing with a cotton swab (15 cm size L, United Medicine Instruments Co., Ltd., Bangkok, Thailand). Then, the cotton swab was inserted into a microtube containing an appropriate solvent solution for each explosive. For 2,4,6-trinitrotoluene (TNT) and 2,4-dinitrotoluene (DNT), the solvent solution was 1:1 acetone–water [4,5]. Deionized water was used as the solvent solution for nitrite and perchlorate. Then, the solution was dropped onto the polymer gel sensors in the plates. Final sample concentrations were 10, 15, 20, 25, and 30 mg L^−1^ for TNT, 0.25, 0.30, 0.35, 0.40, and 0.45 mg mL^−1^ for DNT, 1.5, 2.0, 2.5, 3.0, and 3.5 mg L^−1^ for nitrite, and 0.55, 0.60, 0.65, 0.70, and 0.75 mg mL^−1^ for perchlorate. The recoveries were evaluated according to the AOAC guideline.

## 3. Results and Discussion

### 3.1. Optimization of the Photographic System

**Figure 4:**
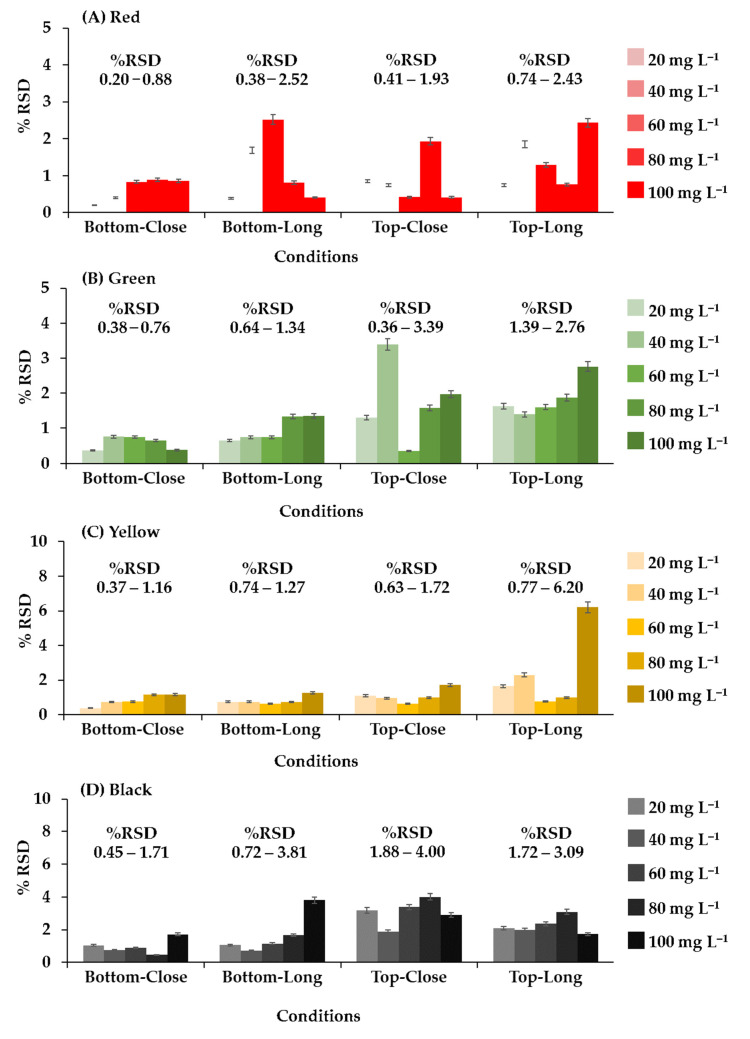
The relative standard deviation of each system configuration from analysis of food coloring; (**A**) red, (**B**) green, (**C**) yellow, and (**D**) black.

The position and distance of the camera relative to the 96-well plate was adjusted, as shown in Figure 4. The system analyzed red, green, yellow, and black food coloring at different concentrations (20, 40, 60, 80, and 100 mg L^−1^) with the camera in each position. In the initial study, food coloring was chosen for the optimum condition of the camera position in the photographic system and the precision (n = 96) of the proposed method because it is inexpensive, nontoxic, harmless, and stable. This constant property eliminates color shifts over time. Different results were obtained from every configuration, but the best results were obtained when the camera was positioned below the plate at a distance of 15 cm (Figure 2A). The relative standard deviations (RSDs) (n = 3) were the lowest, and the RSD ranges were the narrowest at all concentrations and colors of food coloring (0.20–0.88%RSD of red (Figure 4A), 0.38–0.76%RSD of green (Figure 4B), 0.37–1.16%RSD of yellow (Figure 4C), and 0.45–1.71%RSD of black (Figure 4D)). These results showed that the capturing position and the distance between the plate and camera affected the results of analysis. Therefore, in further experiments, images were captured from underneath the 96-well plates at a distance of 15 cm. The relevant AOAC guideline for food coloring specifies an RSD of <5.3% at a concentration of 80 mg L^−1^ [36]. The RSDs of the results of analysis of the entire 96-well plate using the microplate spectrophotometer ranged from 1.67 to 2.20% (Table 1) and therefore were acceptable. The RSDs (n = 96) of the results obtained for the same analysis using the proposed method ranged from 3.24 to 3.61%. The results of the proposed method were acceptable and demonstrated excellent performance and high throughput (96 samples at a time) of the proposed method.

### 3.2. Colorimetric Test of Explosives

#### 3.2.1. Colorimetric Test of TNT and DNT

**Figure 5:**
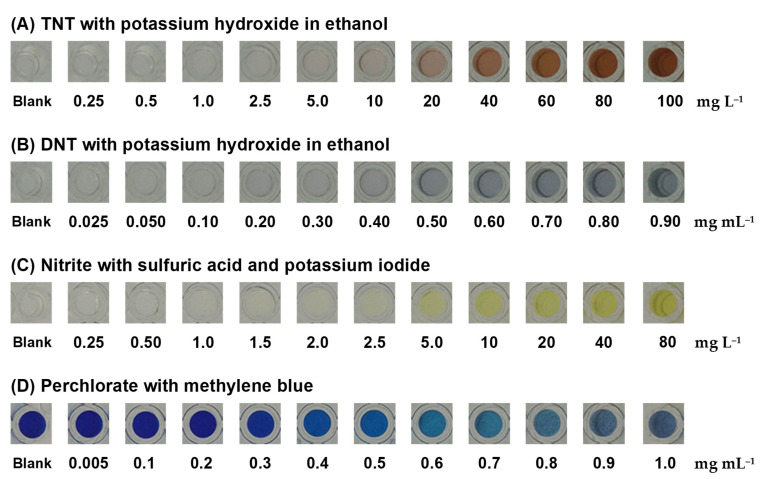
Colorimetric products of (**A**) TNT and (**B**) DNT were produced with potassium hydroxide in ethanol. Colorimetric products of (**C**) nitrite were produced with sulfuric acid and potassium iodide and colorimetric products of (**D**) perchlorate were produced with methylene blue.

A colorimetric test of TNT and DNT with resulting production of a Janowsky complex by the nitro-aromatic rings of TNT and DNT with alkaline acetone was proceeded with potassium hydroxide (KOH) [34,37,38,39]. Clear TNT and DNT standard solutions were added into wells containing KOH in the polymer gel. TNT produced a red-brown product (Figure 5A) and DNT produced a blue product (Figure 5B). The product color intensity increased with increasing TNT or DNT concentrations. By the naked eye, the colored product of 10 mg L^−1^ TNT and 0.5 mg mL^−1^ DNT was visible, indicating that the polymer gel sensor was sensitive enough. These color products were reacted for 5 min prior to capturing the image.

#### 3.2.2. Colorimetric Test of Nitrite

Nitrite reacted with sulfuric acid and potassium iodide in the polymer gel sensor to produce a yellow product of iodine (Figure 5C), and it could produce nitric oxide, potassium sulfate, sodium sulfate, and water, in which the nitrite in the acidic solution selectively reacted with iodide to form iodine [40,41,42,43]. The homogeneous yellow color of the product in the polymer gel sensor intensified with increasing nitrite concentrations, which indicated a homogenous reagent entrapment within the polymer gel matrix.

#### 3.2.3. Colorimetric Test of Perchlorate

Perchlorate was detected through the use of MB in the polymer gel sensor with the precipitation of a violet methylene blue perchlorate complex [44,45,46,47,48]. The increment of the violet precipitate produced by the reaction increased with the concentration of perchlorate, allowing a semiquantitative analysis to be developed. When the concentration of perchlorate was increased, the blue color of MB became lighter, as shown in Figure 5D.

### 3.3. Digital Image Colorimetry for Explosives Quantification

**Figure 6:**
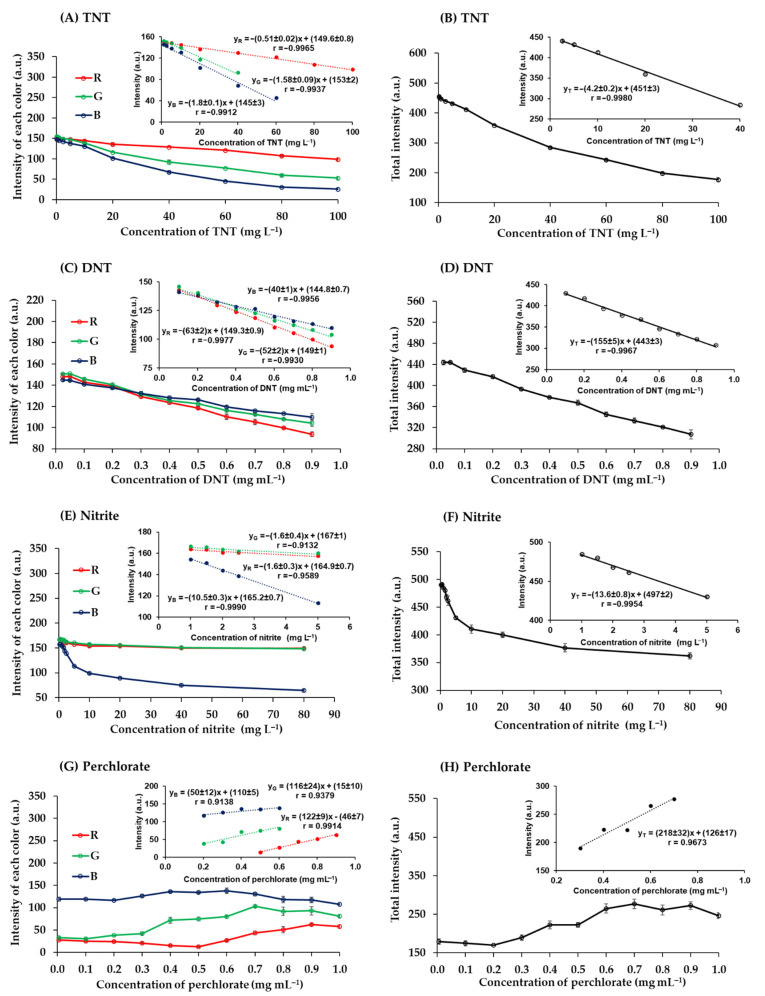
Relationship between the intensity of each color and the concentration of (**A**) TNT, (**C**) DNT, (**E**) nitrite, and (**G**) perchlorate and the relationship between the total intensity and the concentration of (**B**) TNT, (**D**) DNT, (**F**) nitrite, and (**H**) perchlorate.

The quantitative analysis of the studied explosives was achieved using digital image colorimetry. Digital images of color products were analyzed using the RGB analyzer program on a notebook in real time. The program determined RGB intensity values ranging from 0 to 255 for each color. A white image would produce R, G, and B values of 255, 255, and 255, and a black image would produce R, G, and B values of 0, 0, and 0. These images were captured with an industrial video camera within the photographic box. Calibration curves were plotted of color intensities against explosive concentrations (Figure 6).

The red-brown products of TNT reacted with potassium hydroxide were captured and analyzed. The RGB intensity values were correlated with the concentrations of TNT (Figure 6A). The red intensity was higher than green and blue intensities, which is to be expected given the red-brown color of the product, and it remained higher in the concentration range analyzed. The green and blue intensities decreased with increasing TNT concentrations from 0.25 to 100 mg L^−1^, as previously reported. The relationships derived from the combination of the total RGB intensity values (T = R + G + B) (Figure 6B). It was found that the total intensity was a more sensitive measure than the individual RGB values.

The quantification of DNT with potassium hydroxide gave blue products. Blue intensity was lower than red and green intensities at DNT concentrations below 0.3 mg mL^−1^. However, blue intensity was the highest at higher concentrations (Figure 6C) because the products were dark blue [34]. The sensitivity of the total values was higher than the sensitivity of each color intensity (Figure 6D).

Nitrite was quantified by reaction with sulfuric acid and potassium iodide to produce a yellow product. The red and green intensities of this product were similar, while the blue intensity was the lowest at all concentrations of nitrite (0.25 to 80 mg L^−1^) (Figure 6E) because the product was yellow, which is a color combination of red and green [49]. The individual RGB values had a lower sensitivity than the total intensity (Figure 6F).

The quantitative analysis of perchlorate with MB produced a violet precipitate product associated with the MB perchlorate complex. The blue intensity was relatively stable but decreased slightly as the concentration of chlorate increased >0.6 mg mL^−1^ (Figure 6G) due to the increasing presence of the MB perchlorate complex. Conversely, when the concentration of perchlorate increased, the red and green intensity values increased. At perchlorate concentrations from 0.5 to 0.9 mg mL^−1^, red intensity increased more than total RGB intensity, indicating greater sensitivity in this concentration range (Figure 6G,H).

### 3.4. Analytical Performance and Method Validation

The calibration equations, including the linear range and linearity for the quantification of TNT, DNT, nitrite, and perchlorate, are summarized in Table 2. Although the total RGB intensity provided higher sensitivity than the individual RGB values of all explosives (−4.2 ± 0.2 a.u. L mg^−1^ of TNT, −155 ± 5 a.u. mL mg^−1^ of DNT, −13.6 ± 0.8 a.u. L mg^−1^ of nitrite and 218 ± 32 a.u. mL mg^−1^ of perchlorate), the correlation coefficient (r) must be better than 0.995 [50]. Consequently, perchlorate determination was based on the red intensity, and the determination of TNT, DNT, and nitrite was based on the total intensity. The analytical performance of the proposed method was presented in Table 3. The LODs of TNT, DNT, nitrite, and perchlorate were 2.28 mg L^−1^, 0.0523 mg mL^−1^, 0.512 mg L^−1^, and 0.163 mg mL^−1^, respectively. More importantly, even after 30 days of preparation, the polymer gel sensor continued to produce consistent colors for the tests with TNT, DNT, nitrite, and perchlorate. As a result, it was found that the proposed polymer gel sensor has a usable time of at least one month.

### 3.5. Specificity

The specificity of the proposed method toward the four explosives was studied using the explosives singly and in mixtures of two, three, and four explosives (Table 4). The results indicated good specificity. Products of reactions between TNT/DNT and KOH were purple, which is a combination of red/brown and blue. Sulfuric acid/potassium iodide and MB only reacted with nitrite and perchlorate and so could only be used for the qualitative analysis of an explosive mixture to indicate the likely presence of some kind of explosive substance containing nitrite or perchlorate. It should be noted that only nitrite and a mixture of nitrite + perchlorate can be detected. In contrast, the mixed sample containing TNT + nitrite, DNT + nitrite, TNT + DNT + nitrite, TNT + nitrite + perchlorate, DAT + nitrite + perchlorate, and TNT + DNT + nitrite + perchlorate was undetectable. This may be due to decreased nitrite solubility because TNT and DNT use acetone (1:1 acetone–water) as a solvent. The revised manuscript includes these details in Section 3.5. 

### 3.6. Interference

The influence of various foreign species on the determination of the studied multi-explosives using the proposed method was evaluated under the same experimental conditions. The potential interferences comprised urea, Zn^2+^, Ba^2+^, Cu^2+^, Pb^2+^, Ni^2+^, Mg^2+^, Fe^2+^, Sb^3+^, SO_4_^2−^, Cl^−^, and NO_3_^−^. The tolerance limit was assigned as the concentration of the interfering species which caused a relative error of intensity change below ±5% (Table 5). These experimental results suggested that the proposed method provided good selectivity when applied for the simultaneous measurement of TNT, DNT, nitrite, and perchlorate.

### 3.7. Analysis of Case Work Sample

The correlations between the optimal RGB values and the concentration of TNT, DNT, nitrite, and perchlorate was also investigated using six different substrate samples (tables, tile floors, mobile phones, windowsills, PVC pipes, and wall clocks). Percentage recoveries ranged from 83 ± 3 to 106 ± 2% for TNT, from 92 ± 2 to 104 ± 3% for DNT, from 89 ± 2 to 108 ± 2% for nitrite, and from 79.3 ± 0.6 to 118 ± 3% for perchlorate (Table 6). The recoveries of TNT, DNT, and nitrite were acceptable according to AOAC guidelines [36], but perchlorate was slightly above the limit because the violet precipitate product affected the quantitative analysis. The overall results indicated that the proposed method could be applied to qualitative and quantitative screening of multi-explosives.

## 4. Conclusions

The determination of multi-explosives detected with specific polymer gel sensors on 96-well plates was based on the colorimetric analysis of digital images. RGB values were produced from images of reaction products and were successfully applied to quantify the explosive compounds in the reactions. A simple photographic box and a software program were designed to create a portable system that can transmit data wirelessly. The proposed method was rapid (96 samples at a time), accurate, and portable. The real-time analysis enables the use of the method in forensic cases and shows great potential to rapidly progress criminal investigations.

## Figures and Tables

**Table 1:** The relative standard deviations of the proposed method and microplate spectrophotometry for four food colorings at a concentration of 80 mg L^−1^.

Food Coloring	Relative Standard Deviation (n = 96)	
	Microplate Spectrophotometer	Proposed Method
Red	1.78	3.24
Green	1.67	3.61
Yellow	2.04	3.27
Black	2.20	3.59

**Table 2:** The calibration equations for the quantification of the four explosives studied.

Explosive Type	**Relationships**	**Calibration Equation**	Linear Range	r
TNT ^a^	R and C	y_R_ = −(0.51 ± 0.02)x + (149.6 ± 0.8)	1.0–100	−0.9965
	G and C	y_G_ = −(1.58 ± 0.09)x + (153 ± 2)	1.0–40	−0.9937
	B and C	y_B_ = −(1.8 ± 0.1)x + (145 ± 3)	1.0–60	−0.9912
	T and C	y_T_ = −(4.2 ± 0.2)x + (451 ± 3)	2.5–40	−0.9980
DNT ^b^	R and C	y_R_ = −(63 ± 2)x + (149.3 ± 0.9)	0.1–0.9	−0.9977
	G and C	y_G_ = −(52 ± 2)x + (149 ± 1)	0.1–0.9	−0.9930
	B and C	y_B_ = −(40 ± 1)x + (144.8 ± 0.7)	0.1–0.9	−0.9956
	T and C	y_T_ = −(155 ± 5)x + (443 ± 3)	0.1–0.9	−0.9967
Nitrite ^a^	R and C	y_R_ = −(1.6 ± 0.3)x + (164.9 ± 0.7)	1.0–5.0	−0.9589
	G and C	y_G_ = −(1.6 ± 0.4)x + (167 ± 1)	1.0–5.0	−0.9132
	B and C	y_B_ = −(10.5 ± 0.3)x + (165.2 ± 0.7)	1.0–5.0	−0.9990
	T and C	y_T_ = −(13.6 ± 0.8)x + (497 ± 2)	1.0–5.0	−0.9954
Perchlorate ^b^	R and C	y_R_ = (122 ± 9)x − (46 ± 7)	0.5–0.9	0.9914
	G and C	y_G_ = (116 ± 24)x + (15 ± 10)	0.2–0.6	0.9379
	B and C	y_B_ = (50 ± 12)x + (110 ± 5)	0.2–0.6	0.9138
	T and C	y_T_ = (218 ± 32)x + (126 ± 17)	0.3–0.7	0.9673

^a^ unit of mg L^−1^, ^b^ unit of mg mL^−1.^

**Table 3:** Analytical performances of the proposed method from the colorimetric test of the explosives studied.

Explosive Type	**Relationships**	**Sensitivity**	LOD
TNT	T and C	−4.2 ± 0.2 ^a^′	2.28 ^a^
DNT	T and C	−155 ± 5 ^b^′	0.0523 ^b^
Nitrite	T and C	−13.6 ± 0.8 ^a^’	0.512 ^a^
Perchlorate	R and C	122 ± 9 ^b^’	0.163 ^b^

LOD = limit of detection, ^a^ unit of mg L^−1^, ^b^ unit of mg mL^−1^, ^a′^ unit of a.u. L mg^−1^, ^b′^ unit of a.u. mL mg^−1.^

**Table 4:** Specificity of single explosives and multiple combinations of explosives to reagents.

Explosive	Reagent Test		
	KOH	H_2_SO_4_/KI	Methylene Blue
A	+	−	−
B	+	−	−
C	−	+	−
D	−	−	+
A + B	purple (mixed)	−	−
A + C	+	−	−
A + D	+	−	+
B + C	+	−	−
B + D	+	−	+
C + D	−	+	+
A + B + C	purple (mixed)	−	−
A + B + D	purple (mixed)	−	+
A + C + D	+	−	+
B + C + D	+	−	+
A + B + C + D	purple (mixed)	−	+

A = TNT (10 mg L^−1^), B = DNT (0.5 mg mL^−1^), C = Nitrite (2.5 mg L^−1^), D = Perchlorate (0.7 mg mL^−1^), + is positive testing and − is negative testing.

**Table 5:** Tolerance limits of potential interferences on the determination of 10 mg L^−1^ TNT, 0.5 mg mL^−1^ DNT, 2 mg L^−1^ nitrite, and 0.7 mg mL^−1^ perchlorate.

Interference Species	TNT (10 mg ^L−1^)		DNT (0.5 mg ^mL−1^)		Nitrite (NO_2_^−^) (2 mg ^L−1^)		Perchlorate (ClO_4_^−^) (0.7 mg ^mL−1^)	
	Tolerance Ratio	%Relative Error	Tolerance Ratio	%Relative Error	Tolerance Ratio	%Relative Error	Tolerance Ratio	%Relative Error
Urea	600	+2.95	500	−1.73	5000	+2.95	250	−3.51
Zn^2+^	400	−4.76	500	−2.41	5000	+3.50	500	−0.53
Ba2+	200	−2.83	50	−0.71	5000	−3.68	25	−3.30
Cu^2+^	80	+2.46	5	−4.23	400	−1.67	500	−2.34
Pb^2+^	80	−2.90	5	−0.35	50	−4.23	50	−4.21
Ni^2+^	50	+0.37	25	−4.89	5000	−0.94	25	−4.74
Mg^2+^	50	+0.19	500	−0.53	5000	−1.31	100	−4.35
Fe2+	20	−4.53	5	−4.08	150	−4.60	100	−4.89
Sb^3+^	10	+0.81	2	+3.65	500	−1.16	1	−0.73
SO_4_^2−^	400	−4.76	500	−2.41	5000	+3.50	500	−0.53
Cl^−^	200	−2.83	500	−0.53	5000	−3.68	100	−4.35
NO_3_^−^	80	−2.90	5	−0.35	50	−4.23	50	−4.21

**Table 6:** Recoveries from six different substrate samples.

Sample	Explosives											
	TNT (mg L^−1^)			DNT (mg mL^−1^)			Nitrite (mg L^−1^)			Perchlorate (mg mL^−1^)		
	Add	Found	Recovery	Add	Found	Recovery	Add	Found	Recovery	Add	Found	Recovery
1	10	9.8 ± 0.9	98 ± 4	0.25	0.25 ± 0.02	99 ± 3	1.5	1.6 ± 0.1	103.4 ± 0.6	0.55	0.61 ± 0.05	111 ± 4
	15	12.4 ± 0.7	83 ± 3	0.30	0.30 ± 0.01	101 ± 2	2.0	2.0 ± 0.3	100 ± 4	0.60	0.59 ± 0.01	98 ± 1
	20	20.3 ± 0.6	102 ± 3	0.35	0.36 ± 0.01	103 ± 1	2.5	2.4 ± 0.4	98 ± 4	0.65	0.56 ± 0.00	87 ± 0
	25	22 ± 1	89 ± 5	0.40	0.40 ± 0.04	101 ± 5	3.0	3.1 ± 0.4	104 ± 5	0.70	0.58 ± 0.04	83 ± 3
	30	27.2 ± 0.6	91 ± 2	0.45	0.43 ± 0.02	97 ± 3	3.5	3.6 ± 0.2	102 ± 3	0.75	0.73 ± 0.04	97 ± 3
2	10	8.7 ± 0.2	87 ± 1	0.25	0.25 ± 0.02	100 ± 2	1.5	1.5 ± 0.4	98 ± 4	0.55	0.65 ± 0.09	118 ± 3
	15	14.5 ± 0.7	97 ± 3	0.30	0.31 ± 0.03	104 ± 3	2.0	2.0 ± 0.2	99 ± 2	0.60	0.61 ± 0.06	102 ± 4
	20	18 ± 1	90 ± 5	0.35	0.35 ± 0.07	99 ± 8	2.5	2.6 ± 0.3	105 ± 4	0.65	0.62 ± 0.04	96 ± 3
	25	23.0 ± 0.8	92 ± 4	0.40	0.39 ± 0.05	96 ± 6	3.0	3.1 ± 0.4	105 ± 4	0.70	0.59 ± 0.04	84 ± 3
	30	31.8 ± 0.6	106 ± 2	0.45	0.45 ± 0.03	99 ± 4	3.5	3.3 ± 0.1	94 ± 1	0.75	0.61 ± 0.01	81.5 ± 0.6
3	10	8 ± 1	84 ± 5	0.25	0.25 ± 0.01	101 ± 2	1.5	1.6 ± 0.4	106 ± 5	0.55	0.62 ± 0.04	113 ± 3
	15	13 ± 1	86 ± 5	0.30	0.31 ± 0.02	104 ± 2	2.0	2.2 ± 0.3	108 ± 2	0.60	0.64 ± 0.03	106 ± 2
	20	18.3 ± 0.3	91 ± 2	0.35	0.35 ± 0.04	101 ± 4	2.5	2.5 ± 0.1	100.2 ± 0.6	0.65	0.62 ± 0.02	95 ± 2
	25	22.5 ± 0.8	90 ± 4	0.40	0.39 ± 0.05	98 ± 5	3.0	2.9 ± 0.4	96 ± 4	0.70	0.59 ± 0.02	85 ± 2
	30	28.3 ± 0.7	94 ± 3	0.45	0.42 ± 0.05	92 ± 2	3.5	3.6 ± 0.3	102 ± 4	0.75	0.59 ± 0.01	79.3 ± 0.6
4	10	9.6 ± 0.6	91 ± 3	0.25	0.25 ± 0.06	100 ± 7	1.5	1.4 ± 0.2	91 ± 3	0.55	0.62 ± 0.03	112 ± 2
	15	15 ± 1	101 ± 6	0.30	0.31 ± 0.02	102 ± 3	2.0	2.0 ± 0.4	102 ± 4	0.60	0.64 ± 0.02	106 ± 1
	20	18.6 ± 0.2	93 ± 1	0.35	0.36 ± 0.02	104 ± 3	2.5	2.5 ± 0.3	99 ± 4	0.65	0.70 ± 0.1	107.0 ± 0.6
	25	24.0 ± 0.8	96 ± 4	0.40	0.39 ± 0.05	97 ± 7	3.0	3.0 ± 0.2	98 ± 2	0.70	0.72 ± 0.02	103 ± 2
	30	27.1 ± 0.2	90 ± 1	0.45	0.43 ± 0.06	96 ± 7	3.5	3.2 ± 0.1	90.8 ± 0.6	0.75	0.72 ± 0.06	95 ± 4
5	10	9.5 ± 0.9	95 ± 4	0.25	0.25 ± 0.01	102 ± 2	1.5	1.3 ± 0.1	89 ± 2	0.55	0.60 ± 0.05	109 ± 3
	15	15.5 ± 0.2	104 ± 1	0.30	0.30 ± 0.05	102 ± 6	2.0	1.9 ± 0.3	95 ± 4	0.60	0.59 ± 0.03	99 ± 2
	20	19 ± 1	95 ± 6	0.35	0.36 ± 0.03	104 ± 3	2.5	2.6 ± 0.3	104 ± 3	0.65	0.59 ± 0.02	92 ± 2
	25	23.6 ± 0.7	94 ± 3	0.40	0.41 ± 0.01	104 ± 2	3.0	3.1 ± 0.2	103 ± 3	0.70	0.66 ± 0.04	94 ± 3
	30	28.4 ± 0.9	94 ± 4	0.45	0.45 ± 0.04	100 ± 5	3.5	3.6 ± 0.3	104 ± 3	0.75	0.60 ± 0.08	81 ± 6
6	10	10 ± 1	103 ± 5	0.25	0.24 ± 0.03	96 ± 3	1.5	1.5 ± 0.3	102 ± 3	0.55	0.60 ± 0.05	109 ± 3
	15	13.0 ± 0.9	87 ± 4	0.30	0.31 ± 0.04	103 ± 5	2.0	1.9 ± 0.3	94 ± 3	0.60	0.64 ± 0.04	106 ± 3
	20	17.1 ± 0.9	85 ± 4	0.35	0.34 ± 0.03	96 ± 3	2.5	2.4 ± 0.2	96 ± 3	0.65	0.71 ± 0.05	109 ± 4
	25	22.6 ± 0.6	91 ± 3	0.40	0.40 ± 0.02	101 ± 3	3.0	3.0 ± 0.4	100 ± 5	0.70	0.80 ± 0.08	114 ± 6
	30	27.7 ± 0.6	92 ± 3	0.45	0.45 ± 0.02	100 ± 2	3.5	3.6 ± 0.2	102 ± 3	0.75	0.74 ± 0.09	99 ± 7

## Data Availability

The data presented in this study are available on request from the corresponding author.
